# Comparative Physiological and Transcriptomic Analyses Reveal Altered Fe-Deficiency Responses in Tomato Epimutant *Colorless Non-ripening*

**DOI:** 10.3389/fpls.2021.796893

**Published:** 2022-01-21

**Authors:** Wei Wei Chen, Hui Hui Zhu, Jia Yi Wang, Guang Hao Han, Ru Nan Huang, Yi Guo Hong, Jian Li Yang

**Affiliations:** ^1^Research Centre for Plant RNA Signaling, College of Life and Environmental Sciences, Hangzhou Normal University, Hangzhou, China; ^2^State Key Laboratory of Plant Physiology and Biochemistry, College of Life Sciences, Zhejiang University, Hangzhou, China

**Keywords:** tomato *Cnr*, Fe deficiency, Fe-deficiency responses, comparative transcriptome, Fe-deficiency-responsive genes

## Abstract

The mechanisms associated with the regulation of iron (Fe) homeostasis have been extensively examined, however, epigenetic regulation of these processes remains largely unknown. Here, we report that a naturally occurring epigenetic mutant, *Colorless non-ripening* (*Cnr*), displayed increased Fe-deficiency responses compared to its wild-type Ailsa Craig (AC). RNA-sequencing revealed that a total of 947 and 1,432 genes were up-regulated by Fe deficiency in AC and *Cnr* roots, respectively, while 923 and 1,432 genes were, respectively, down-regulated. Gene ontology analysis of differentially expressed genes showed that genes encoding enzymes, transporters, and transcription factors were preferentially affected by Fe deficiency. Kyoto Encyclopedia of Genes and Genomes pathway enrichment analysis revealed differential metabolic responses to Fe deficiency between AC and *Cnr*. Based on comparative transcriptomic analyses, 24 genes were identified as potential targets of *Cnr* epimutation, and many of them were found to be implicated in Fe homeostasis. By developing CRISPR/Cas9 genome editing *SlSPL-CNR* knockout (KO) lines, we found that some *Cnr*-mediated Fe-deficiency responsive genes showed similar expression patterns between *SlSPL-CNR* KO plants and the *Cnr* epimutant. Moreover, both two KO lines displayed Fe-deficiency-induced chlorosis more severe than AC plants. Additionally, the *Cnr* mutant displayed hypermethylation in the 286-bp epi-mutated region on the *SlSPL-CNR* promoter, which contributes to repressed expression of *SlSPL-CNR* when compared with AC plants. However, Fe-deficiency induced no change in DNA methylation both at the 286-bp epi-allele region and the entire region of *SlSPL-CNR* gene. Taken together, using RNA-sequencing and genetic approaches, we identified Fe-deficiency responsive genes in tomato roots, and demonstrated that *SlSPL-CNR* is a novel regulator of Fe-deficiency responses in tomato, thereby, paving the way for further functional characterization and regulatory network dissection.

## Introduction

Iron (Fe) is an essential microelement for higher plants, involved in many physiological and metabolic processes such as photosynthesis, substance metabolism, and respiration. Although Fe is abundantly present in the earth’s crust, its bioavailability is quite low due to the insoluble form of ferric (Fe^3+^) hydroxide in alkaline soils that occupy approximately 30% of the arable land worldwide (Guerinot and Yi, 1994). On the other hand, excess Fe is toxic to living organisms due to the production of reactive hydroxyl radicals (Halliwell and Gutteridge, 1992). Therefore, plants have evolved complicated regulatory mechanisms to control Fe homeostasis.

When suffering from Fe deficiency, two strategies have been developed for higher plants to acquire Fe from rhizosphere. One is the reduction-based mechanism in dicot and non-graminaceous monocot (Strategy I), and the other is the chelation-based mechanism in graminaceous monocot (Strategy II) (Kobayashi and Nishizawa, 2012). In Strategy I plants, such as *Arabidopsis* and tomato, Fe^3+^ is first reduced by a plasma membrane-localized Ferric Reduction Oxidase 2 (FRO2) (Robinson et al., 1999), and then transported across the membrane into cells by Iron-regulated Transporter 1 (IRT1) (Vert et al., 2002). However, Strategy II plants such as maize, rice, and barley secrete phytosiderophores to chelate Fe^3+^, which is then transported across the membrane by oligopeptide transporter Yellow-stripe 1 (YS1) in maize and its functional homolog OsYSL15 in rice (Curie et al., 2001; Inoue et al., 2009). In addition, Fe^3+^ solubilization is mediated by both H^+^-ATPase-dependent rhizosphere acidification and secretion of Fe^3+^-mobilizing coumarins (Santi and Schmidt, 2009; Tsai and Schmidt, 2017).

Intricate regulatory networks of gene expression controlled by transcription factors (TFs) is crucial for Fe-deficiency responses in plants. In Arabidopsis, Fe-efficiency reactions (FER)-like iron deficiency-induced TF (FIT) is the master regulator characterized to be implicated in the Fe homeostasis (Colangelo and Guerinot, 2004). While FIT, a basic helix–loop–helix (bHLH) TF, is transcriptionally regulated by Fe deficiency, it can interact with the subgroup Ib bHLH TFs to form heterodimers, which in turn activate the expression of *IRT1* and *FRO2* (Yuan et al., 2008). Interestingly, the transcripts of subgroup Ib bHLH TFs are also modulated by Fe deficiency and their transcription is directly modulated by subgroup IVc bHLH TFs (Zhang et al., 2015; Li et al., 2016; Liang et al., 2017). Recently, it was established that another bHLH TF, bHLH121, or URI, acts upstream of the Fe homeostasis network by interacting with subgroup IVc bHLH TFs to bind to the promoters of subgroup Ib bHLH TFs except FIT to activate their expression under Fe deficiency (Kim et al., 2019; Gao et al., 2020). In addition, protein phosphorylation has been identified to be involved in post-translational modification of FIT activity (Gratz et al., 2019, 2020).

To avoid toxicity with excess Fe, plants have developed feedback regulatory pathways to control Fe homeostasis. A pericycle-specific Fe-deficiency responsive bHLH TF, POPEYE (PYE), helps maintain Fe homeostasis by regulating the expression of Fe-homeostasis-related genes including *NAS4*, *FRO3*, and *ZIF1* (Long et al., 2010). A member of hemerythrin E3 ligases, BRUTUS (BTS), functions as a putative Fe sensor and interacts with subgroup IVc bHLH TFs to mediate their degradation through 26S proteasome-induced ubiquitination, which in turn negatively regulates Fe-deficiency responses (Kobayashi et al., 2013; Selote et al., 2015). In the absence of ethylene or nitric oxide (NO), FIT was previously identified to be severely degraded *via* 26S proteasome (Lingam et al., 2011; Meiser et al., 2011), the pathway has been recently proposed to be mediated by two partially redundant E3 ubiquitin ligases, BRUTUS-LIKE1 (BTSL1) and BTSL2 (Rodríguez-Celma et al., 2019). Furthermore, a RING E3 ubiquitin ligase, IRT1 degradation factor1 (IDF1), ubiquitinates IRT1 to regulate its degradation, thus allowing plants to respond promptly to altering environmental Fe conditions (Shin et al., 2013).

As an important commercial vegetable worldwide, changes in metabolic processes have been studied in tomato under Fe deficiency, where organic acids especially citrate production has been observed (López-Millán et al., 2009). It has been reported that Fe deficiency can induce NO accumulation, which functions downstream of auxin in inducing root branching to enhance Fe-deficiency tolerance in tomato cultivar Micro-Tom (Jin et al., 2011). However, so far, little is available for molecular basis of Fe-deficiency responses in tomato, although the first cloned regulatory gene characterized in Fe homeostasis was FER, a bHLH TF from tomato (Ling et al., 2002), and subsequently another five tomato bHLH TFs participated in Fe-deficiency responses have been reported (Sun et al., 2015).

*Colorless non-ripening* (*Cnr*) is a spontaneous epigenetic mutant, which performs normal growth and development, but produces fruits that cannot ripen and remain colorless. Genetic analysis indicated that the phenotype is caused by hypermethylated cytosines in a recessive allele at *SlSPL-CNR* locus (Manning et al., 2006). However, a recent CRISPR/cas9 genome editing of *SlSPL-CNR* failed to recreate the complete lack of ripening phenotype in the *Cnr* epimutant, questioning the requirement of SlSPL-CNR during fruit ripening (Gao et al., 2019). Nonetheless, our previous study found that SlSPL-CNR acts as a negative regulator by directly binding to the promoter of nitrate reductase (NR) to suppress its transcription and NR-mediated NO accumulation, which then contributes to Cadmium (Cd) uptake *via IRT1* expression and enhanced Cd sensitivity in the *Cnr* epimutant (Chen et al., 2018b). Considering NO and IRT1 are important for Fe-deficiency responses as well, we were wondering whether and how *Cnr* epimutation is responsible for Fe-deficiency responses in tomato. In this study, *via* physiological and transcriptomic analyses of the *Cnr* epimutant upon Fe deficiency, we demonstrated that *SlSPL-CNR* epimutation is responsible for Fe-deficiency responses, and genes implicated in altered Fe-deficiency responses in the *Cnr* epimutant were identified. Collectively, we provided molecular bases of Fe-deficiency response mediated by *Cnr* epimutation in tomato.

## Materials and Methods

### Plant Materials and Growth Conditions

In this study, wild-type (WT) tomato [*Solanum lycopersicum* cv. Ailsa Craig (AC)] and the *Cnr* epimutant on the AC background (Manning et al., 2006; Chen et al., 2015a,b, 2018a,b) were used. Two independent *SlSPL-CNR* knockout lines were generated from tomato transformation *via* the CRISPR/Cas9-induced genome editing system.

All tomato seeds were surface-sterilized and germinated on plates containing 1/5 Hoagland nutrient solution (pH 5.5) and 1% (w/v) agar in a greenhouse under controlled conditions (Chen et al., 2018b). The 1/5 Hoagland nutrient solution has the following composition (in μM): Ca(NO_3_)_2_ (1,000), KNO_3_ (1,000), MgSO_4_ (400), NH_4_H_2_PO_4_ (200), H_3_BO_3_ (3), ZnSO_4_ (0.4), CuSO_4_ (0.2), MnCl_2_ (0.5), (NH_4_)_6_(Mo_7_) (1), and FeEDTA (20), with pH 5.5 adjusted by 1 M KOH. After germination, when the primary roots were approximately 3-4 cm in length, uniform seedlings were transplanted to aerated hydroponics with 1/5 Hoagland nutrient solution. Two days later, the acclimatized seedlings were transferred to 1 L pots that contained media either with 20 mM FeNaEDTA (+Fe) or without iron (–Fe) for another 3 or 9 days. The treatment solution was refreshed every other day. Roots were harvested in liquid nitrogen and stored at −80°C for RNA isolation.

### CRISPR/Cas9-Induced Knock Out of *SlSPL-CNR* in Tomato

To generate *SlSPL-CNR* knockout transgenic plants, the online program CRISPR-PLANT^1^ was used to select two specific sgRNAs that targeted the first exon and second intron on the genomic DNA of SlSPL-CNR, respectively. The selected sgRNAs were then PCR-amplified and fused into the CRISPR/Cas9-mediated knockout vector to generate pSlSPL-CNR-KO construct as previously described (Zhang et al., 2017). The recombinant vector was confirmed by Sanger sequencing and then introduced into AC by stable *Agrobacterium*-mediated leaf disk transformation as described by Yao et al. (2020). In brief, AC cotyledons were prepared and incubated in the *Agrobacterium tumefaciens* GV3101 containing pSlSPL-CNR-KO construct. After that, cotyledon explants were transferred to a selection medium for callus induction and shoot regeneration. Subsequently, regenerated shoots were separated from the original explants and transferred to a rooting medium for root development. For mutations induced by Cas9, genomic DNA was isolated from young leaves of transgenic lines, and PCR and Sanger sequencing were performed using primers flanking the target sites. Transgene-free plants were screened and double confirmed in the T1 and T2 generations. Two independent homozygous *SlSPL-CNR* knockout lines named as KO1 and KO2 were selected for further analysis. For the off-target effects assay, nine putative off-target sites (Supplementary Table 3) were examined using the Cas-OFFinder^2^ (Bae et al., 2014), and PCR products obtained from the edited plants were sequenced. The gene-specific primers used are listed in Supplementary Table 1.

### Determination of Fe-Deficiency-Induced Chlorosis and Chlorophyll Synthesis

To explore the leaf chlorosis induced by Fe deficiency, seedlings of AC, *Cnr*, and KO lines were treated under +Fe or –Fe conditions, and then photographically recorded with a Nikon digital camera. For chlorophyll content determination, newly formed leaves were collected from AC, *Cnr*, and KO lines under +Fe or –Fe treatment, and then embedded in 80% acetone overnight in darkness with internal shaking every 4 h until the leaves are colorless. Subsequently, the absorbance values of the extracts were spectrophotometrically measured at 663 and 645 nm, and the total chlorophyll in each sample was recorded as chlorophyll content per gram fresh weight (FW), which was in accordance with a modified method as previously described (Robinson and Wellburn, 1991).

### Root Morphological Analysis and Root Ferric Chelate Reductase Assay

After 7 days of Fe-deficient treatment, the whole tomato roots were excised and photographed by a Nikon digital camera. Meanwhile, observation of the root tip and the relative maturation zone patterns was performed by a CCD camera (Nikon Eclipse Ni microscope).

Measurement of the subsequent ferric chelate reductase (FCR) activity was performed based on a reported procedure (Grusak, 1995; Chen et al., 2010). Briefly, the whole tomato roots were excised and immersed in the FCR assay solution [0.5 mM CaSO_4_, 0.1 mM MES, 0.1 mM BPDS, and 100 mM Fe-EDTA (pH 5.5)] at room temperature (approximately 25°C). After incubation for 1 h in dark, the absorbance of assay solutions was determined at 535 nm using a spectrophotometer (Bio-Rad, United States). FCR localization along the roots was carried out as previously described (Chen et al., 2010). The excised roots were embedded in plates containing 0.5 mM CaSO_4_, 0.5 mM FeNaEDTA, and 0.5 mM ferrozine (pH 5.5), which was solidified with 0.75% (w/v) agarose. Roots were incubated in a dark room at 25°C for 20 min, and then the color patterns were photographed using a digital camera and a Nikon Eclipse Ni microscope with transmitted-light detector.

### *In situ* Measurement of Nitric Oxide Level in Tomato Roots

*In situ* NO level was determined by staining with DAF-FM DA according to our previous studies (Chen et al., 2010, 2018b). Briefly, root tips were loaded with 10 μM DAF-FM DA in 20 mM HEPES/NaOH buffer (pH 7.4) for 30 min, and then NO fluorescence was recorded microscopically using a Nikon epi-fluorescence microscope (Nikon Eclipse Ni, Japan). NO signal intensities of green fluorescence in the images were quantified using Photoshop software (Adobe Systems) as previously described (Guo and Crawford, 2005). Data are shown as the mean of fluorescence intensity relative to that of AC under Fe sufficiency.

### RNA Extraction and Quantitative RT-PCR Analysis

Total RNA was extracted and cDNAs were synthesized using FastQuant RT Kit with gDNA Eraser (Tiangen). The quantitative reverse-transcription PCR (RT-qPCR) was carried out on a LightCycler480 machine (Roche Diagnostics, Switzerland) using SYBR Green chemistry (Toyobo) with sequence-specific primers (Supplementary Table 1). The relative level of specific gene expression was calculated using the ^ΔΔ^Ct method, which was normalized against the internal control *ACTIN* mRNA detected in the same sample as described previously (Chen et al., 2018b).

### Transcriptome RNA-Sequencing

For RNA-sequencing, tomato seedlings of AC and *Cnr* epimutant were transferred into the Fe-sufficient (+Fe) or Fe-deficient (–Fe) media for 3 days, and then the whole root was harvested for RNA isolation. Approximately 5 μg pooled RNA was used for TrueSeq library construction, and RNA-sequencing was performed on an Illumina HiSeq Platform using the PE150 mode. Three biological replicates were performed for each treatment in repeated experiments. The high-quality reads from the RNA-sequencing raw data were mapped to the tomato reference genome (version SL2.50)^3^ using Bowtie2 (Langmead and Salzberg, 2012), and differentially expressed genes (DEGs) were analyzed using DEGseq2 as described (Love et al., 2014). RNA-sequencing data are available as accession number PRJNA681103 in the NCBI SRA database.^4^

### DNA Extraction and Targeted-Bisulfite Sequencing

DNeasy Plant Mini Kit (Qiagen) was used to isolate the genomic DNA from tomato roots harvested from AC and *Cnr* epimutant under +Fe or –Fe treatment for 3 days. After DNA integrity was checked by agarose-gel electrophoresis, approximately 500 ng of purified genomic DNA was bisulfite-converted using the EZ DNA Methylation-Gold kit (Zymo Research) according to the manufacturer’s protocol. The following target bisulfite-sequencing PCR for the entire gene region of *SlSPL-CNR*, *SlIRT1;1*, *SlIRT1;2*, *SlbHLH066*, *SlS8H*, *Ring finger protein 38*, and *SlPIN5* was carried out using the Double PCR mixture (CoWin Bioscience) with gene-specific primers (Manning et al., 2006; Supplementary Table 1). The methylation information of cytosine (C) at various sites was collected for further in-house bioinformatics analyses (Manning et al., 2006; Chen et al., 2015b).

### Statistical Analysis

Each experiment was carried out independently at least two times, and the resulting data given as mean ± standard deviation (SD) were represented of at least ten biological replicates for FCR activity, NO accumulation, and chlorophyll content assays, while at least three biological replicates for transcriptional expression assays were related to RNA-sequencing and specific gene expression. All statistical analyses were performed by Tukey’s test with *P* ≤ 0.05 considered to be statistically significant among different treatments (Tang and Zhang, 2013).

## Results

### *Colorless Non-ripening* Epimutant Displayed Increased Responses to Fe Deficiency

**FIGURE 1:**
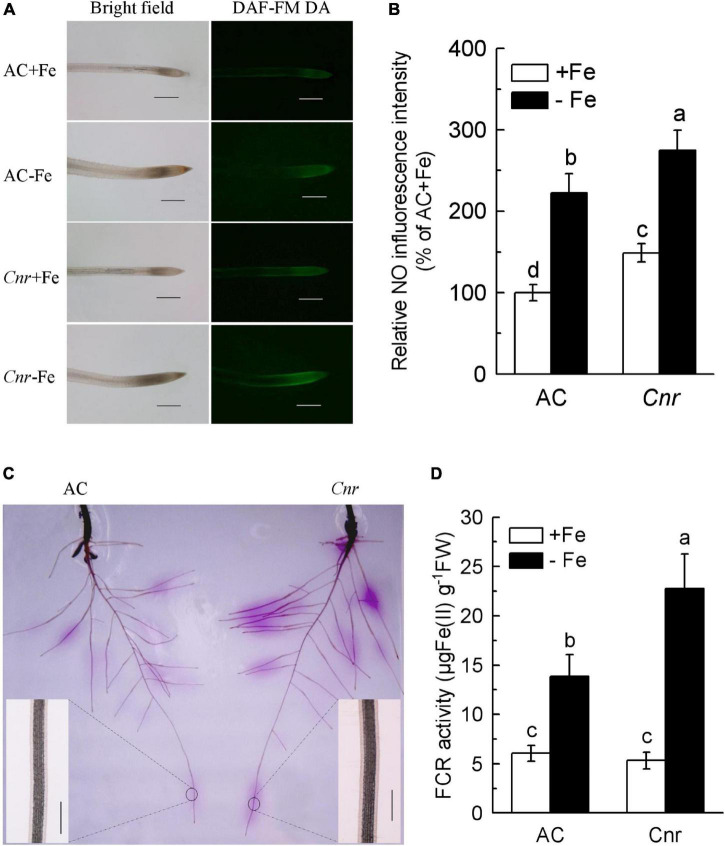
Increased Fe-deficiency responses in *colorless non-ripening* (*Cnr*) roots compared to AC plants. **(A)** Examination of endogenous nitric oxide (NO) levels. Uniform seedlings of AC and *Cnr* were subjected to Fe-sufficient (+Fe) or Fe-deficient (–Fe) conditions for 3 days. At least 10 seedlings were examined for each treatment and the representative photos were given. Bar = 500 μm. **(B)** Quantification of the NO fluorescence intensity using Adobe Photoshop software. **(C)** In-gel localization of Fe-deficiency-induced root ferric chelate reductase (FCR) activity. Enlarged inlet images show detailed localization patterns of FCR along root tip under –Fe conditions. Bar = 500 μm. **(D)** The induction of FCR activity under Fe deficiency. Data are means ± SD (*n* = 6). Different letters indicate significant differences between AC and *Cnr* under +Fe or –Fe treatment (Tukey’s test, *P* ≤ 0.05).

It was previously found that a naturally occurring epigenetic mutant, *Cnr*, produced more NO in root apex compared to AC plants (Chen et al., 2018b). Since NO has been widely documented as being implicated in the Fe-deficiency response in plants (Romera et al., 2011), we were wondering whether this epigenetic mutation is responsible for Fe-deficiency response. Thus, NO production was determined using an NO-specific fluorescence probe DAF-FM DA (Figure 1A). Under Fe-sufficient condition, the fluorescence intensity in *Cnr* root apex was greater than that in AC. In comparison with their respective Fe-sufficient control roots, Fe deficiency induced the fluorescence intensity to increase by 2.22- and 1.85-fold in AC and *Cnr* roots, respectively. Interestingly, endogenous NO concentration was much greater in the *Cnr* epimutant than that in AC under Fe deficiency (Figure 1B). However, no obvious differences were observed in Fe-deficiency-induced chlorosis, chlorophyll content, as well as root morphology between AC and *Cnr* epimutant (Supplementary Figure 1). Since the induction of root FCR activity has been proved to be a rate-limiting step for Fe uptake in Strategy I plants (Connolly et al., 2003), root FCR activity was examined in both AC and *Cnr* plants under Fe deficiency. Notably, a significant enhancement of FCR activity was found both in AC and *Cnr* roots under Fe deficiency, and this Fe-deficiency-induced FCR activity was much higher in *Cnr* than that in AC (Figures 1C,D), indicating that *Cnr* is more sensitive to Fe deficiency than AC. These results suggest that *Cnr* epimutation is implicated in the Fe-deficiency responses in tomato.

### Global Effects of Fe Deficiency on Gene Expression in Tomato Roots

To understand the underlying molecular bases of *Cnr*-dependent Fe-deficiency response in tomato, transcriptome analysis was carried out in both AC and *Cnr* roots subjected to either Fe-sufficient or Fe-deficient conditions for 3 days. As shown in Supplementary Tables 4, 12 samples were subjected to RNA-sequencing, and produced approximately 6.42 Gb data for each sample. The average genome mapping rate and average gene mapping rate were 91.46 and 77.58%, respectively. With three biological replicates for each treatment, the correlation coefficients showed acceptable reproducibility, indicating the high reliability of our RNA-sequencing data (Supplementary Figure 2).

Next, clean reads were aligned to the genome sequence using Bowtie2 (Langmead and Salzberg, 2012), and gene expression levels of each sample were calculated using RSEM (Li and Dewey, 2011). The DEGs were identified with log2-fold change (FC) ≥ 1 or log2 FC ≤ -1 and with *Q*-value (adjusted *P*-value) < 0.05 by DEseq2 (Love et al., 2014). Thereby we identified 947 up-regulated (Supplementary Table 5) and 923 down-regulated (Supplementary Table 6) genes in AC roots under Fe-deficiency condition (Supplementary Figures 3A,B). In contrast, a total of 1,432 up-regulated (Supplementary Table 7) and 1,852 down-regulated (Supplementary Table 8) genes were identified to be affected by Fe deficiency in *Cnr* roots (Supplementary Figures 3A,B).

Gene ontology (GO) analysis of DEGs showed that in both AC and *Cnr*, genes assigned to “cellular process,” “metabolic process,” and “response to stimuli” are highly enriched in biological processes (Supplementary Figures 3C,D). In the cellular component category, genes belonging to “membrane,” “membrane part,” and “cell” are most abundant. In the molecular function category, the highly enriched genes are implicated in “catalytic activity,” “binding,” and “transporter activity.” These results indicate that genes encoding enzymes, transporters, and transcription factors were preferentially affected by Fe deficiency, consisting of a biological function of Fe as cofactors of enzymes. Enrichment analysis of KEGG pathways showed that genes involved in phenylpropanoid biosynthesis, glycolysis/gluconeogenesis, and photosynthesis were most represented in AC roots (Supplementary Figure 4A). However, genes involved in glutathione metabolism, phenylpropanoid biosynthesis, and carbon metabolism were the most significantly enriched in *Cnr* roots (Supplementary Figure 4B). While these results suggest the possible pathways related to Fe-deficiency response in tomato, metabolic responses to Fe deficiency differed between AC and *Cnr*.

### Identification of Gene Homologs Associated With Fe-Deficiency Response in Tomato

Whilst substantial progress has been made toward identifying and characterizing genes implicated in Fe-deficiency response in *Arabidopsis*, little is known about how tomato responds to Fe deficiency at the genome-wide level. Therefore, we identified tomato genes homologous to known Fe-deficiency-responsive genes in *Arabidopsis* (Supplementary Table 9). This included genes encoding transcription factors, transporters, coumarin biosynthesis, Fe storage, and others. Among TFs, we found that, similar to Arabidopsis, the transcription level of FER and subgroup Ib bHLH TFs were induced by Fe deficiency, while genes encoding subgroup IVc bHLH TFs, that is, SlbHLH104, SlbHLH115, and SlILR3, were mostly not responding to Fe deficiency at the transcriptional level, with exception of SlILR3. Two genes homologous to *Arabidopsis* ILR3 were identified. While one (Solyc07g064040) was not transcriptionally responding to Fe deficiency, the other (Solyc07g052670) was induced. Furthermore, we identified 16 transporter genes implicated in Fe uptake, translocation from roots to shoots, and internal mobilization, whose expression was mostly observed to be differentially regulated by Fe deficiency. However, genes involved in Fe storage, that is, genes encoding ferritin and the vacuolar iron transporter family proteins, were detected to be mostly down-regulated by Fe deficiency. These results provided a base for future functional characterization of genes related to Fe-deficiency response in tomato.

In a previous study, we found that the expression of *SlNR* (Solyc11g013810) was higher in *Cnr* than AC (Chen et al., 2018b). Here, in our RNA-sequencing data, we also found that the expression level of *SlNR* was induced by 1.76-fold in *Cnr* roots by comparison with AC under the Fe-sufficient condition (Supplementary Tables 5, 6). Considering nitrate reductase is one of the pathways for NO production in plants and NO was found to be more abundant in *Cnr* under Fe-sufficient conditions, we were interested to know whether *SlNR* is responsible for Fe-deficiency-induced NO production. While the expression of *SlNR* was found to be highly expressed in *Cnr* roots than that in AC roots, Fe deficiency resulted in the down-regulation of *SlNR* expression in AC, although its expression was slightly up-regulated in the *Cnr* epimutant (Supplementary Tables 5, 6), suggesting that *SlNR* is not involved in NO production in response of tomato to Fe deficiency. This result is consistent with a previous report that nitric oxide synthase (NOS) but not NR is involved in NO accumulation under Fe deficiency in tomato (Jin et al., 2011).

### Identification of *Colorless Non-ripening*-Mediated Fe-Deficiency-Responsive Genes in Tomato

**FIGURE 2:**
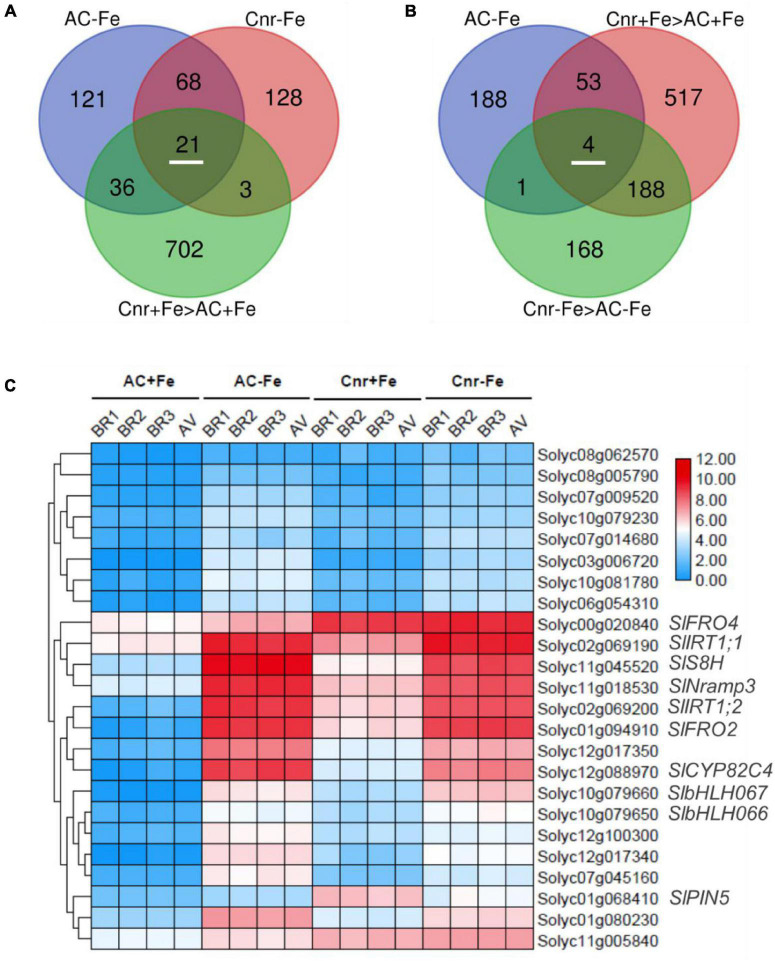
Identification of potential *Cnr*-mediated Fe-deficiency-responsive genes. **(A)** Venn diagram shows 21 Fe-deficiency-responsive genes whose expression could be constitutively induced by *Cnr* epimutation under Fe-sufficient condition. **(B)** Venn diagram shows 4 *Cnr*-mediated Fe-deficiency-responsive genes whose expression was higher in *Cnr* epimutant than that in AC under Fe-deficient condition. **(C)** Expression profiles of potential *Cnr*-mediated Fe-deficiency-responsive genes. BR, biological replicate; AV, average value.

To find *Cnr*-mediated Fe-deficiency responsive genes, we performed comparative transcriptome analyses between AC and *Cnr* with two Fe conditions (+Fe and –Fe). To this end, we extracted DEGs whose expression was induced by more than 2-folds (log_2_ FC ≥ 1 and *Q*-value < 0.05). Under such threshold, there are, respectively, 220 and 246 genes significantly induced by Fe deficiency in *Cnr* (*Cnr*-Fe) and AC (AC-Fe) (Supplementary Tables 4, 5). Since the *Cnr* epimutant displayed an increased response to Fe deficiency when in comparison with AC plants (Figure 1), *Cnr* epimutation could be related to the increase of gene expression. Among 89 Fe-deficiency responsive genes that overlapped between AC and *Cnr*, 21 showed induced expression in the *Cnr* epimutant under Fe-sufficient condition (Figure 2A), which were regarded as constitutively *Cnr*-induced Fe-deficiency-responsive genes. Interestingly, many of these genes are involved in Fe homeostasis. This included genes that are implicated in Fe uptake such as Solyc02g069200 (*SlIRT1;1*), Solyc02g069190 (*SlIRT1;2*), and Solyc01g094910 (*SlFRO2*), and genes that are implicated in Fe mobilization such as Solyc12g088970 (*cytochrome P450 82C4*), Solyc11g045520 (*SlS8H*), and *SlNramp3*, and genes related to transcription regulation such as *SlbHLH066* and *SlbHLH067* involved in interacting with as well as activating FER. In line with these results, our previous report indicated that the *Cnr* epimutant resulted in the constitutive up-regulation of *SlIRT1* by comparison with AC plants (Chen et al., 2018b).

**FIGURE 3:**
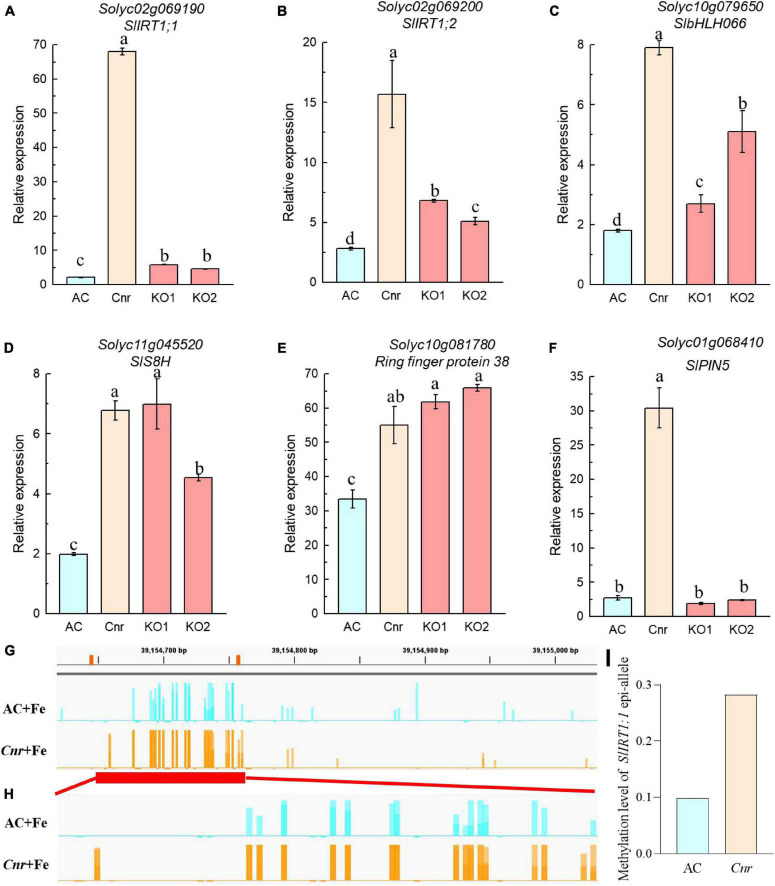
Expression analysis of *Cnr*-mediated Fe-deficiency-responsive genes by RT-qPCR. **(A–F)** Roots of AC, *Cnr*, and two *SlSPL-CNR* knockout plants (KO1 and KO2) grown in Fe-sufficient solution were harvested for RNA extraction and qRT-PCR analysis. *ACTIN* was used as an internal control to normalize expression. Data are means ± SD (*n* = 3 for biological repeats). Different letters indicate significant differences among AC, *Cnr* and two KO lines under +Fe treatment by Tukey’s test at *P* ≤ 0.05 level. **(G,H)** Targeted bisulfite sequencing reveals changes of DNA methylation at the *SlIRT1;1* gene promoter **(G)** and the 115-bp epi-allele region of *SlIRT1;1* promoter **(H)** in AC and *Cnr* roots after 3 days under +Fe condition, respectively. **(I)** The methylation levels of the epi-allele in the promoter region of *SlIRT1;1* as shown in panel **(H)**.

Next, we identified genes whose expression could be further enhanced by *Cnr* epimutation under Fe deficiency. Among 762 *Cnr*-epimutation-regulated genes under Fe-sufficient condition (Cnr + Fe > AC + Fe), 57 genes were regulated by Fe deficiency in AC roots, and the expression of 4 genes could be further induced by Fe deficiency in *Cnr* roots (Figure 2B). They are Solyc01g068410 homologous to Arabidopsis PIN5, Solyc11g005840 coding for cysteine desulfurase, Solyc00g020840 homologous to Arabidopsis FRO4, and Solyc08g062570 coding for glutathione S-transferase. It is noteworthy that Solyc08g062570 was observed in both analyses. Therefore, a total of 24 genes were found to be the potential targets of *Cnr* epimutation (Figure 2C). GO analysis showed that these genes are mainly related to ion transport, membrane, and ion binding in biological process, cellular component, and molecular function, respectively (Supplementary Figure 5). Furthermore, to confirm that the expression regulation of these Fe-deficiency-responsive genes is truly dependent on *Cnr* epimutation, RT-qPCR analysis was performed to validate their differential expression under Fe-sufficient condition, and 6 out of 24 genes were selected to find that the expression of all the six genes was higher in the *Cnr* epimutant than that in AC plants (Figures 3A–F), suggesting that our RNA-sequencing and gene identification are highly reliable.

### Is *SlSPL-CNR* Responsible for *Colorless Non-ripening*-Mediated Fe-Deficiency Response?

**FIGURE 4:**
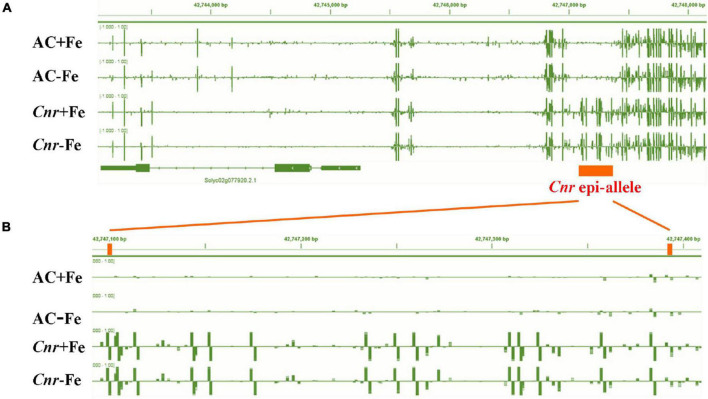
Fe deficiency induced no significant changes in DNA methylation at the overall region of *SlSPL-CNR* gene in *Cnr* roots. Targeted bisulfite sequencing reveals changes of DNA methylation at the overall region of *SlSPL-CNR* gene **(A)** and the 286-bp epi-allele region of *SlSPL-CNR* promoter **(B)** in AC and *Cnr* roots after 3 days under +Fe or –Fe condition. Bar-chart shows the methylation levels in the *SlSPL-CNR* gene locus. The location of the *Cnr* epi-allele in the promoter region of *SlSPL-CNR* is shown.

**FIGURE 5:**
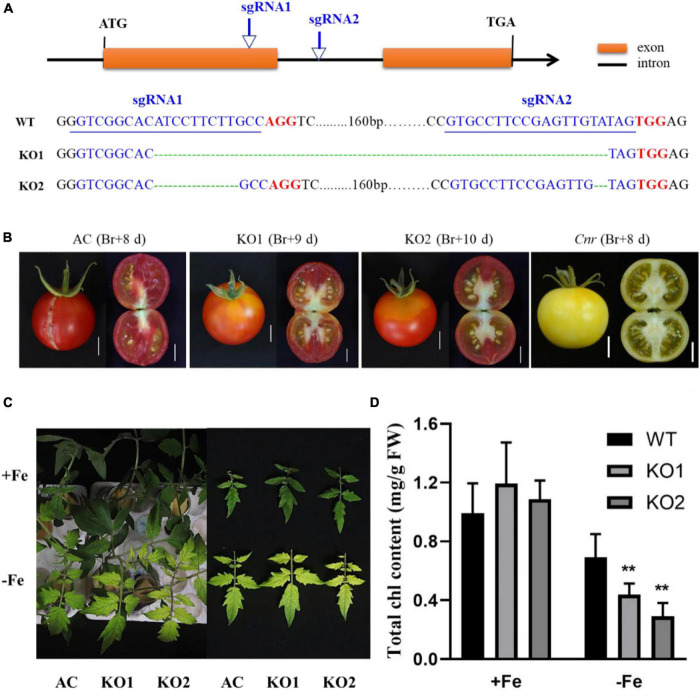
Construction of *SlSPL-CNR* knockout (KO) mutants and relative physiological responses under Fe-deficiency. **(A)** Schematic illustration of CRISPR/Cas9-mediated mutation pattern in *SlSPL-CNR* gene. The two sgRNAs target sites in *SlSPL-CNR* coding sequence (138–160 bp for sgRNA1, 325–347 bp for sgRNA2), related PAM sites and CRISPR edited sites are represented in blue, red, and green, respectively. *Via* Sanger sequencing, KO1 displayed a frame-shift type mutation with 196-bp deletion located at 146–341 bp between two target sites, while KO2 displayed an in-frame type mutation with 9-bp deletion located in the first target site and 2-bp deletion located in the second target site, respectively. **(B)** Fruit phenotypes of AC, two independent KO lines KO1 and KO2. Br, break. Bar = 1 cm. **(C)** Fe-deficiency-induced chlorosis in newly formed leaves of AC and two KO lines after 9 days under +Fe or –Fe condition. **(D)** Chlorophyll content in newly formed leaves of AC and two KO lines. A significant reduction of chlorophyll levels was seen in two KO lines when compared with AC under Fe deficiency (Tukey’s test, *P* ≤ 0.05, as indicated by asterisks). Data are shown as means ± SD (*n* = 4 for biological repeats).

A previous study suggested that the colorless non-ripening phenotype of the *Cnr* epimutant was ascribed to the hypermethylation of a 286-bp promoter region, resulting in a decrease of LeSPL-CNR expression in the fruits (Manning et al., 2006). This prompted us to investigate whether increased Fe-deficiency responses in *Cnr* are connected with the DNA methylation changes in *Cnr* roots. Targeted-bisulfite sequencing was used to investigate the effect of Fe deficiency on cytosine methylation in the promoter and gene body region of both *SlSPL-CNR* and 6 selected potential *Cnr*-mediated Fe-deficiency-responsive genes. There was no significant change in methylation on the overall region of *SlSPL-CNR* gene (including 286-bp epi-mutated region on its promoter in both AC and *Cnr* roots under Fe deficiency compared with that under Fe sufficiency (Figure 4A). It is noteworthy that, in comparison to AC, *Cnr* exhibited highly methylated cytosine residues in the 286-bp epiallele region of the *SlSPL-CNR* promoter regardless of Fe status (Figure 4B), which contribute to the inhibition of the *SlSPL-CNR* expression in *Cnr* (Manning et al., 2006). However, there were no discernable differences in DNA methylation between AC and *Cnr* for all 6 selected genes except *SlIRT1;1* (Supplementary Figure 6 and Supplementary Table 2). In the 115-bp epiallele region of the *SlIRT1;1* promoter, *Cnr* showed a higher methylation level than AC under Fe-sufficient condition (Figures 3G–I), suggesting that DNA methylation changes are unlikely to be directly related to altered transcriptional changes of these six genes. Therefore, we wanted to know whether *SlSPL-CNR* is attributable to *Cnr*-mediated Fe-deficiency responses in tomato roots. For this purpose, two specific target sites, sgRNA1 and sgRNA2, were designed for *SlSPL-CNR* gene, and two independent transgene-free lines were generated using the CRISPR/Cas9 system (Figure 5A). There is a large DNA fragment deleted including parts of both sgRNAs and 160 bp DNA sequence between two sgRNAs in KO1, while there are 9 bp deletion in sgRNA1 and 2 bp deletion in sgRNA2 in KO2 (Figure 5A). Moreover, no mutations were detected in all nine potential off-target sites, suggesting that mutagenesis generated at the designed target sites is of high specificity (Supplementary Table 3). Consistent with a previous report (Gao et al., 2019), two *SlSPL-CNR* KO lines were found to only display delayed ripening phenotype in comparison to AC, which is in contrast to *Cnr* epimutant (Figure 5B).

Next, we compared the expression of *Cnr*-mediated Fe-deficiency-responsive genes in *Cnr* to that of *SlSPL-CNR* KO lines by RT-qPCR. While the expression levels of *SlS8H* (Solyc11g045520) and *Ring finger protein 38* (Solyc10g081780) in both KO lines were similar to those of *Cnr* epimutant, the expression of *SlPIN5* (Solyc01g068410) was not affected by *SlSPL-CNR* impairment in comparison with AC plants. On the other hand, the expression of *SlIRT1;1* (Solyc02g069190), *SlIRT1;2* (Solyc02g069200), and *SlbHLH066* (Solyc10g079650) was higher in both KO lines than AC plants, although their expression remains significantly lower than *Cnr* epimutant (Figure 3), implying that SlSPL-CNR might be partially responsible for *Cnr*-mediated Fe-deficiency responses in tomato.

To further confirm the involvement of *SlSPL-CNR* in Fe-deficiency responses, we compared Fe-deficiency-induced chlorosis between AC and two KO lines. After treatment for 9 days, although no significant difference in chlorophyll content was observed among AC and two KO lines under Fe-sufficient condition, the newly formed leaves were more severely chlorotic with less chlorophyll content in both KO lines when compared with AC plants (Figures 5C,D), which was in agreement with the greater up-regulation of Fe-deficiency-responsive genes in the *Cnr* epimutant under Fe-deficient condition.

## Discussion

The tomato *Cnr* is a naturally occurring epimutant, containing hypermethylated cytosines in the promoter region of an SBP box TF, which was thought to be associated with the colorless non-ripening phenotype of fruits (Manning et al., 2006). A previous report, however, suggested that many ripening gene promoters became hypermethylated in *Cnr*, suggesting that *Cnr* epimutation must have a wider effect on the epigenome of tomato (Zhong et al., 2013). In this study, we reported the involvement of *Cnr* in Fe-deficiency responses in tomato. This is supported by the findings that *Cnr* produced more NO compared to AC plants (Figure 1A), which has been regarded as an important signaling molecule implicated in triggering Fe-deficiency response in plants (Chen et al., 2010; Yang et al., 2013). Consistently, we previously found that *Cnr* was more sensitive to Cd stress than AC plants due to intensified accumulation of NO (Chen et al., 2018b). Furthermore, *Cnr* roots displayed a higher activity of FCR than AC roots under Fe-deficiency condition (Figure 1C). Finally, the expression of some featured Fe-deficiency-responsive genes was induced in the *Cnr* epimutant under Fe-sufficient condition (Figure 2). Our comparative transcriptome analyses revealed that there are 24 genes whose transcription levels were increased in the *Cnr* mutant (Figure 2). It is noteworthy that many of these genes are known to be crucial for Fe homeostasis in plants (Supplementary Figure 5), that is, SlFRO2 is responsible for the reduction of Fe at the root surface, and SlIRT1 is the major Fe^2+^ transporter. Therefore, *Cnr* epimutation has pleiotropic effects on both development and stress response.

The available evidence suggests that epigenetic modifications such as histone modification and DNA methylation play important roles in plants responding to nutrient stresses (Secco et al., 2017). For example, extensive global remodeling of DNA methylation associated with gene expression has been observed upon Pi starvation in Arabidopsis (Yong-Villalobos et al., 2015). Several members of the histone deacetylase (HDAC) family were found to be implicated in both cell length and expression regulation of some Pi-responsive genes under Pi-deficient condition (Chen C. Y. et al., 2015). Recently, a histone deacetylase complex1 (HDC1) was reported to be negatively involved in Pi-starvation response in Arabidopsis and the loss-of-function mutant *hdc1* showed increased sensitivity to Pi starvation (Xu et al., 2020). The regulatory roles of histone posttranslational modifications in Fe homeostasis have been previously reported. For example, Fan et al. (2014) reported that Shk1-binding protein1-mediated histone arginine 3 (H4R3) demethylation negatively regulates Fe homeostasis by affecting the expression of some subgroup Ib bHLH genes and Fe-uptake processes. GENERAL CONTROL NON-REPRESSED PROTEIN5 (GCN5) mediates histone3 lysine 9 and lysine 14 acetylation of FRD3, which promotes its transcription activation, and consequently Fe translocation from roots to shoots (Xing et al., 2015). PRC2-mediated H3K27me3 (histone 3 lysine 27 trimethylation) modulated the expression of FIT-dependent genes under iron deficiency (Park et al., 2019). Recently, *NRF2/ELF8* controls the expression of the root-specific gene *GRF11* through H3K4me3 and maintains the Fe-uptake machinery (Singh et al., 2021). Taking into account that the *Cnr* epimutant displayed increased sensitivity to Fe-deficiency response (Figure 1), it is interesting to envision whether epigenetic modifications, especially DNA methylation, play potential roles in Fe-deficiency responses.

Considering that the phenotype of *Cnr* fruits is ascribed to DNA hypermethylation of many ripening gene promoters, it is reasonable to deduce that differential expression patterns of *Cnr*-mediated Fe-deficiency-responsive genes are associated with DNA methylation changes. The study from tomato fruits demonstrated that CHROMOMETHYLASE3 (CMT3) responsible for methylation maintenance at CHG context is required for the somatic inheritance of the *Cnr* epimutant (Chen et al., 2015b), provided further evidence that *Cnr* epimutation is closely associated with DNA methylation change. However, it is worth noting that, in fruits, *Cnr* epimutation was frequently related to DNA hypermethylation which is typically associated with inactive transcription (Zhong et al., 2013; Chen et al., 2015b). Indeed, we also found that the *Cnr* epimutation contributes to DNA hypermethylation similar to that in the promoter of *SlIRT1;1*, but the hypermethylation was associated with active transcription contrary to the regulation partners in *Cnr* fruits (Figure 3). Interestingly, *Cnr* epimutation did result in hypermethylation at the 286-bp epi-allele region of *SlSPL-CNR* promoter in Fe-sufficient roots, however, Fe deficiency induced no significant changes in DNA methylation both at 286 bp epi-mutated region and the entire *SlSPL-CNR* gene (Figure 4), implying that the induction of some Fe-deficiency-responsive genes in the *Cnr* epimutant might be the secondary effects of *Cnr* epimutation. Therefore, it is of importance to perform the methylome analysis to further reveal the epigenetic mechanisms of *Cnr*-mediated Fe-deficiency response in tomato.

The colorless immature phenotype of *Cnr* has previously ascribed to the expression depression of *SlSPL-CNR* (Manning et al., 2006). However, mutation of *SlSPL-CNR via* the CRISPR/Cas9 genome editing showed that *SlSPL-CNR* has a minor role in controlling fruit ripening (Gao et al., 2019). By developing the similar knockout mutant *via* the CRISPR/Cas9 genome editing technique, we also found that functional impairment of *SlSPL-CNR* failed to phenocopy *Cnr* (Figure 5B). Thus, a similar question arises on the role of *SlSPL-CNR* in mediating the expression of Fe-deficiency-responsive genes. In this study, we demonstrated that *SlSPL-CNR* might be in part responsible for *Cnr*-mediated expression regulation of Fe-deficiency-responsive genes. Our RT-qPCR analysis revealed that the expression profiles of some *Cnr*-mediated genes were similar between *Cnr* and *SlSPL-CNR* knockout lines (Figure 3). In addition, *SlSPL-CNR* knockout lines displayed more severe chlorosis than AC plants under Fe deficiency (Figures 5C,D). It seems that *SlSPL-CNR* could function as a transcriptional suppressor because the expression of *SlS8H* was upregulated in both knockout lines. This suggestion was also supported by the previous report that *SlSPL-CNR* can bind to the promoter of *SlNR* and repress its transcription (Chen et al., 2018b).

Although tomato is a model plant for fruit development, less attention was paid to nutrient stress responses by using tomato as a model. Bioinformatic studies have focused on the identification of gene families in response to diverse biotic and abiotic stresses. For example, we previously characterized NAC TF gene family and Acyl-activating enzyme gene family in tomato in response to Al stress (Jin et al., 2020, 2021). Sun et al. (2015) analyzed *bHLH* gene family and identified several *bHLH* gene homologs to *Arabidopsis* bHLH Fe-deficiency-responsive genes. However, transcriptome analysis of tomato under Fe deficiency has not yet been reported. In the present study, we identified genes potentially implicated in tomato Fe-deficiency responses according to *Arabidopsis* homologous gene analysis (Supplementary Table 9). These results for the first time pave the way for future functional characterization and regulatory network dissection of Fe-deficiency signaling in tomato.

In summary, we revealed that a naturally occurring epigenetic mutant *Cnr* showed an increased response to Fe deficiency, as evidenced by the accumulation of NO, greater FCR activity, and enhanced gene expression. Furthermore, we identified potential target genes of the *Cnr* epimutant by comparative transcriptome analysis. We proposed that *Cnr* epimutation resulted in the inhibition of *SlSPL-CNR* that represses the expression of genes related to Fe homeostasis, thereby enhancing Fe-deficiency responses. Altogether, we provided molecular bases of Fe-deficiency responses in tomato, which will be helpful for in-depth functional characterization of genes critical for Fe-deficiency response in tomato.

## Data Availability Statement

The original contributions presented in the study are publicly available. This data can be found here: National Center for Biotechnology Information (NCBI) BioProject database under accession number PRJNA681103.

## Author Contributions

WC and HZ performed the experiments and carried out the data analysis. JW, GH, and RH were involved in bioinformatics analysis. JY and YH initiated and conceived this study, analyzed the data, and wrote the manuscript. All authors read and approved the final manuscript.

## Conflict of Interest

The authors declare that the research was conducted in the absence of any commercial or financial relationships that could be construed as a potential conflict of interest.

## Publisher’s Note

All claims expressed in this article are solely those of the authors and do not necessarily represent those of their affiliated organizations, or those of the publisher, the editors and the reviewers. Any product that may be evaluated in this article, or claim that may be made by its manufacturer, is not guaranteed or endorsed by the publisher.

## Footnotes

1
http://crispr.hzau.edu.cn/CRISPR/

2
http://www.rgenome.net/cas-offinder/

3
https://solgenomics.net

4
https://www.ncbi.nlm.nih.gov/bioproject/
