# Supplementary material for: Comparative Physiological and Transcriptomic Analyses Reveal Altered Fe-Deficiency Responses in Tomato Epimutant Colorless Non-ripening
Source: Front Plant Sci. 2022 Jan 21;12:796893. doi: 10.3389/fpls.2021.796893 (PMC8813752; doi:10.3389/fpls.2021.796893)
Supplement: Supplementary file 1 [file Data_Sheet_1.docx]

**Supplemental Figure S1**


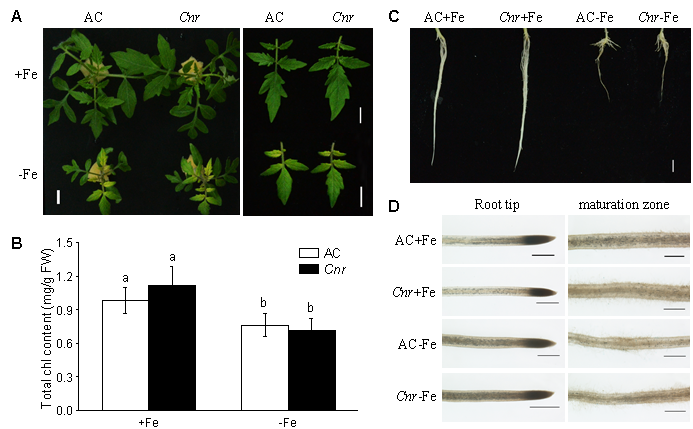


**Supplementary Figure S1.** Fe-deficiency induced physiological responses in AC and *Cnr* epi-mutant. **(A-B)** Fe-deficiency induced chlorosis and the relative chlorophyll content in the newly formed leaves of AC and *Cnr* epi-mutant under +Fe or -Fe treatment for 7 d. Bar = 1 cm in panel (A). Data are shown as means ± SD (n = 7 for biological repeats) in panel (B) with different letters indicating significant differences between AC and *Cnr* under +Fe or –Fe treatment (Tukey’s test, P ≤ 0.05). **(C-D)** Phenotype of whole root, root tip and the maturation zone of root tip in AC and *Cnr* under +Fe or -Fe treatment for 7 d. Bar = 1 cm in panel (C), whilst bar = 500 µm in panel (D).
