# Supplementary material for: Comparative Physiological and Transcriptomic Analyses Reveal Altered Fe-Deficiency Responses in Tomato Epimutant Colorless Non-ripening
Source: Front Plant Sci. 2022 Jan 21;12:796893. doi: 10.3389/fpls.2021.796893 (PMC8813752; doi:10.3389/fpls.2021.796893)
Supplement: Supplementary file 2 [file Data_Sheet_2.docx]

**Supplemental Figure S2**

**Supplementary Figure S2.** The correlations of RNA-sequencing biological replicates of AC and *Cnr*.
