# Supplementary material for: Comparative Physiological and Transcriptomic Analyses Reveal Altered Fe-Deficiency Responses in Tomato Epimutant Colorless Non-ripening
Source: Front Plant Sci. 2022 Jan 21;12:796893. doi: 10.3389/fpls.2021.796893 (PMC8813752; doi:10.3389/fpls.2021.796893)
Supplement: Supplementary file 3 [file Data_Sheet_3.docx]

## Supplemental Figure 3

**Supplementary Figure 3.** Number of differentially expressed genes (DEGs) induced by Fe deficiency and Gene ontology (GO) enrichment analysis of DEGs. **(A)** Up-regulated DEGs in AC and *Cnr* roots. **(B)** Down-regulated DEGs in AC and *Cnr* roots. **(C-D)** GO enrichment analysis of DEGs in AC and *Cnr* epimutant.
