# Supplementary material for: Comparative Physiological and Transcriptomic Analyses Reveal Altered Fe-Deficiency Responses in Tomato Epimutant Colorless Non-ripening
Source: Front Plant Sci. 2022 Jan 21;12:796893. doi: 10.3389/fpls.2021.796893 (PMC8813752; doi:10.3389/fpls.2021.796893)
Supplement: Supplementary file 4 [file Data_Sheet_4.docx]

## Supplemental Figure 4

**Supplementary Figure 4.** Kyoto Encyclopedia of Genes and Genomes (KEGG) enrichment analysis of DEGs in AC (A) and Cnr (B) roots responding to Fe deficiency.
