# Supplementary material for: Comparative Physiological and Transcriptomic Analyses Reveal Altered Fe-Deficiency Responses in Tomato Epimutant Colorless Non-ripening
Source: Front Plant Sci. 2022 Jan 21;12:796893. doi: 10.3389/fpls.2021.796893 (PMC8813752; doi:10.3389/fpls.2021.796893)
Supplement: Supplementary file 5 [file Data_Sheet_5.docx]

## Supplemental Figure 5

**Supplementary Figure 5.** GO category analysis of 24 *Cnr*-mediated Fe-deficiency-responsive Genes.
