# Supplementary material for: Comparative Physiological and Transcriptomic Analyses Reveal Altered Fe-Deficiency Responses in Tomato Epimutant Colorless Non-ripening
Source: Front Plant Sci. 2022 Jan 21;12:796893. doi: 10.3389/fpls.2021.796893 (PMC8813752; doi:10.3389/fpls.2021.796893)
Supplement: Supplementary file 6 [file Data_Sheet_6.docx]

## Supplemental Figure 6

**Supplementary Figure 6.** Targeted bisulfite sequencing reveals changes of DNA methylation at the overall region of *SlIRT1;1* (Solyc02g069190), *SlIRT1;2* (Solyc02g069200), *SlbHLH066* (Solyc10g079650), *SlS8H* (Solyc11g045520), *Ring finger protein 38* (Solyc10g081780) and *SlPIN5* (Solyc01g068410) in AC and *Cnr* roots under +Fe condition for 3 d. Bar-chart shows the methylation levels in the gene locus.
